# Maternal alcohol consumption and risk of offspring with congenital malformation: the Japan Environment and Children’s Study

**DOI:** 10.1038/s41390-020-01274-9

**Published:** 2020-11-23

**Authors:** Hiroshi Kurita, Noriko Motoki, Yuji Inaba, Yuka Misawa, Satoshi Ohira, Makoto Kanai, Teruomi Tsukahara, Tetsuo Nomiyama, the Japan Environment and Children’s Study (JECS) Group, Michihiro Kamijima, Shin Yamazaki, Yukihiro Ohya, Reiko Kishi, Nobuo Yaegashi, Koichi Hashimoto, Chisato Mori, Shuichi Ito, Zentaro Yamagata, Hidekuni Inadera, Takeo Nakayama, Hiroyasu Iso, Masayuki Shima, Youichi Kurozawa, Narufumi Suganuma, Koichi Kusuhara, Takahiko Katoh

**Affiliations:** 1 Center for Perinatal, Pediatric, and Environmental Epidemiology, Shinshu University School of Medicine, Matsumoto, Nagano Japan; 2 Department of Dentistry and Oral Surgery, Shinshu University School of Medicine, Matsumoto, Nagano Japan; 3 Department of Neurology, Nagano Children’s Hospital, Azumino, Nagano Japan; 4 Department of Preventive Medicine and Public Health, Shinshu University School of Medicine, Matsumoto, Nagano Japan; 5 Department of Rehabilitation, Nagano Children’s Hospital, Azumino, Nagano Japan; 6 Department of Obstetrics and Gynecology, Shinshu University School of Medicine, Matsumoto, Nagano Japan; 7 Nagoya City University, Nagoya, Japan; 8 National Institute for Environmental Studies, Tsukuba, Japan; 9 National Center for Child Health and Development, Tokyo, Japan; 10 Hokkaido University, Sapporo, Japan; 11 Tohoku University, Sendai, Japan; 12 Fukushima Medical University, Fukushima, Japan; 13 Chiba University, Chiba, Japan; 14 Yokohama City University, Yokohama, Japan; 15 University of Yamanashi, Chuo, Japan; 16 University of Toyama, Toyama, Japan; 17 Kyoto University, Kyoto, Japan; 18 Osaka University, Suita, Japan; 19 Hyogo College of Medicine, Nishinomiya, Japan; 20 Tottori University, Yonago, Japan; 21 Kochi University, Nankoku, Japan; 22 University of Occupational and Environmental Health, Kitakyushu, Japan; 23 Kumamoto University, Kumamoto, Japan

## Abstract

**Background:**

The association between fetal exposure to alcohol and congenital structural disorders remains inconclusive. The present study searched for relationships between maternal alcohol consumption during pregnancy and the risk of congenital malformations.

**Methods:**

We evaluated the fixed dataset of a large national birth cohort study including 73,595 mothers with a singleton live birth. Information regarding the alcohol consumption of mothers was obtained from self-reported questionnaires. Physicians assessed for 6 major congenital malformations (congenital heart defects [CHDs], male genital abnormalities, limb defects, cleft lip and/or cleft palate [orofacial clefts (OFC)], severe brain abnormalities, and gastrointestinal obstructions) up to 1 month after birth. Multiple logistic regression analysis was performed to identify associations between maternal alcohol consumption during pregnancy and each malformation.

**Results:**

The prevalence of maternal drinking in early pregnancy and until the second/third trimester was 46.6% and 2.8%, respectively. The onset of CHD was inversely associated with mothers who quit drinking during early pregnancy (OR 0.85, 95% CI 0.74–0.98). There was no remarkable impact of maternal drinking habit status on the other congenital malformations after adjustment for covariates.

**Conclusions:**

Maternal alcohol consumption during pregnancy, even in early pregnancy, displayed no significant adverse impact on congenital malformations of interest.

**Impact:**

- This large-scale Japanese cohort study revealed that no teratogenic associations were found between maternal retrospective reports of periconceptional alcohol consumption and congenital malformations after adjustment for covariates.
- This is the first nationwide birth cohort study in Japan to assess the effect of maternal alcohol consumption during pregnancy on major congenital malformations.
- Our finding indicated that maternal low-to-moderate alcohol consumption during pregnancy, even in early pregnancy, displayed no significant adverse impact on congenital heart defects, male genital abnormalities, limb defects, orofacial clefts, severe brain abnormalities, or gastrointestinal obstructions.

## Introduction

Congenital anomalies, such as structural and functional disorders, may result from genetic or chromosomal disorders, exposure to medications or chemicals, or certain infections during pregnancy. Several other risk factors have been reported, including a folate deficiency, alcohol consumption or smoking during pregnancy, poorly controlled diabetes, maternal age >35 years, and socioeconomic status.^1,2^ Fetal alcohol spectrum disorder is a well-known disorder that results from moderate to excessive alcohol exposure during gestation. Maternal alcohol consumption during pregnancy causes brain abnormalities, central nervous system dysfunction, and growth deficiencies of forming organs and body systems.^3^ The most prominent effects of prenatal alcohol exposure are on the developing brain and their associated cognitive and behavioral alterations.^3,4^ Maternal alcohol consumption is also thought to contribute to several other congenital abnormalities, including congenital heart defects (CHDs); cleft lip and/or cleft palate (orofacial clefts (OFC)); congenital limb deficiencies; and anomalies of the kidney, liver, and gastrointestinal tract.^3,5,6^ However, the precise relationship between maternal alcohol consumption during pregnancy and these congenital structural disorders remains inconclusive.

The aim of the present study was to assess for independent associations between maternal alcohol consumption during pregnancy and the risk of common congenital structural disorders and malformations after adjusting for potential confounding risk factors in a large population-based nationwide birth cohort study.

## Methods

### Study design and participants

The data used in this study were obtained from the Japan Environment and Children’s Study (JECS), an ongoing cohort study that commenced in January 2011 to evaluate the effects of environmental factors on child health.

In the JECS, pregnant women were recruited between January 2011 and March 2014. The selection criteria for participants were as follows: (1) residence in the study area at the time of recruitment, (2) expected delivery after August 1, 2011, and (3) ability to comprehend the Japanese language and complete the self-administered questionnaire. The JECS project has been reported previously.^7,8^ The present study used the “jecs-an-20180131” dataset released in March 2018 that included information regarding 98,255 mothers who had a singleton live birth. After registration, the mothers’ data were collected using self-reported questionnaires during the first trimester (MT1) and during the second-third trimester (MT2). These mothers’ questionnaire collected information on demographic factors, medical and obstetric history, physical and mental health, lifestyle, occupation, environmental exposure at home and in the workplace, housing conditions, and socioeconomic status. The perinatal medical records, including pregnancy details and children’s information at birth and 1 month after delivery, were obtained from medical transcripts that were completed by physicians or nurses and used for other covariates.

The Institutional Review Board on Epidemiological Studies of the Ministry of the Environment and the Ethics Committees of all participating institutions (the National Institute for Environmental Studies that leads the JECS, Asahikawa Medical College, Chiba University, Doshisha University, Fukushima Medical University, Hokkaido University, Hyogo College of Medicine, Japanese Red Cross Hokkaido College of Nursing, Kochi University, Kumamoto University, Kyoto University, Kyushu University, Nagoya City University, Osaka Medical Center and Research Institute for Maternal and Child Health, Osaka University, Sapporo Medical University, Shinshu University, the National Center for Child Health and Development, Tohoku University, Tottori University, University of Miyazaki, University of Occupational and Environmental Health, University of Ryukyu, University of Toyama, University of Yamanashi, and Yokohama City University) approved the JECS protocol. All JECS procedures were performed in accordance with tenets set forth by the Helsinki Declaration and other nationally valid regulations and guidelines. All participants provided written informed consent.

### Loss to follow-up

Most of the questionnaires during pregnancy were distributed to women attending prenatal examinations, with some sent by post. Completed questionnaires were submitted during subsequent prenatal visits or mailed. When possible, respondents who gave incomplete answers were interviewed face to face or by telephone for missing details. The numbers of responses from the participants for the MT1 and MT2 questionnaires at baseline are described in a previous study.^7^ The total number of registered pregnancies was 103,099. The response rates of the MT1 and MT2 questionnaires were 96.8 and 95.1%, respectively. The mean (standard deviation) gestational ages at the time of the MT1 and MT2 questionnaire responses were 16.4 (8.0) and 27.9 (6.5) weeks, respectively.^9^ In the “jecs-an-20180131” dataset, the response rates for alcohol consumption, maternal educational status, and annual household income were 96.8, 97.1, and 90.7%, respectively. Regarding the medical records of mothers in early pregnancy and children at birth, the response rates were 100%.^10^

### Data collection

Information regarding the alcohol consumption habits of mothers was obtained during the second/third trimester of pregnancy from the questionnaires along with data on the socioeconomic status and smoking habits of mothers and their partner. Maternal anthropometric data before pregnancy; complications and medication during pregnancy that included placental abnormalities, hypertensive disorders of pregnancy (HDP), and diabetes mellitus/gestational diabetes mellitus (DM/GDM); and history of previous pregnancy were collected via medical record transcripts. Pre-pregnancy body mass index (BMI) to assess maternal weight status was calculated as body weight (kg)/height (m)^2^ according to World Health Organization standards.

### Outcomes, exposure, and covariates

The main outcomes of interest were congenital malformations diagnosed by physicians up to 1 month after birth. We selected quantifiable diseases that had a relatively high prevalence or that could be diagnosed because they are symptomatic or visually identified at birth: CHDs, male genital abnormalities such as hypospadia and cryptorchidism; limb defects, such as polydactyly, syndactyly, and cleft finger/foot; OFC; brain abnormalities, including hydrocephalus, anencephaly, and holoprosencephaly; and gastrointestinal obstructions, including esophageal, duodenal, and small intestinal atresia; and imperforate anus. We removed cases of such chromosomal abnormalities as trisomy 21, trisomy 18, trisomy 13, and Turner syndrome. Subjects with congenital malformations other than the above outcomes of interest or who were complicated by ≥2 congenital malformations were excluded as well.

We examined the data regarding drinking habits as self-reported by participants during the second/third trimester (M2), described as follows: (1) no alcohol consumption, (2) quit drinking before pregnancy, (3) quit drinking during early pregnancy, and (4) currently drinking. Regarding maternal drinking status, mothers were divided into 3 groups: non-drinkers (answer selection 1 or 2), early drinkers (answer selection 3), and current drinkers who continued drinking until second/third trimester of pregnancy (answer selection 4) (Supplemental Tables S1 and S2, online).

The subjects identifying as (4) were further asked to report the frequency, type, and amount of alcohol. Alcohol consumption was evaluated with a semi-quantitative food frequency questionnaire, which included a list of foods and beverages along with the standard portion sizes generally consumed in Japan.^11^ Respondents reporting alcohol consumption during pregnancy were asked on the frequency and amount of what drinks they had. Maternal drinking frequency was evaluated by the questionnaire item: “Please select the response that best describes your current drinking frequency.” The selections were categorized as follows: “hardly ever drink,” “once to three times a month,” “once or twice a week,” “three or four times a week,” “five or six times a week,” or “drink every day.” Alcohol content values for each beverage (Japanese sake, Japanese distilled spirits, beer, whiskey, and wine) were added to determine the total exposure amount of ethanol (g/week). We estimated that 180 mL of Japanese sake contained 23 g of ethanol, 180 mL of distilled spirits contained 36 g of ethanol, a large bottle of beer (633 mL) contained 23 g of ethanol, 30 mL of whiskey contained 10 g of ethanol, and 60 mL of wine contained 9 g of ethanol. Drinkers were classified into low (<1.5 drinks/week) and high (1.5+ drinks/week) absolute alcohol amount categories.^12^ The low alcohol amount group included respondents who indicated that they hardly ever drank. One standard drink was defined to contain 14 g of ethanol.^12,13^ Non-drinking mothers were analyzed as comparison references.

Maternal age, pre-pregnancy BMI, smoking habit of the mothers and her partner, and socioeconomic status including the highest level of education completed by the mother (junior high school, high school, vocational school/junior college, or university/graduate school) and annual household income were employed as demographic covariates. Obstetric and medical variables, such as means of pregnancy, and complications during the gestational period, including DM/GDM and HDP, and intrauterine fetal infection, were also assessed. In the present study, covariates were based on previously published literature and biologic plausibility.^1,2,14–18^

### Statistical analysis

Statistical analyses were carried out using the SPSS statistical software version 24 (SPSS Inc., Chicago, IL). Maternal age and pre-pregnancy BMI were compared among the types of congenital malformations by one-way repeated measures of analyses of variance (ANOVA) followed by post hoc (Bonferroni) testing. All continuous and ordinal variables, such as maternal age (<35 or 35+ years), pre-pregnancy BMI (<18.5, 18.5–24.9, or 25+ kg/m^2^), and annual household income (<4,000,000, 4,000,000–7,999,999, or 8,000,000+ JPY) were categorized. ANOVA and chi-square tests were performed to compare covariates between groups classified by category as well as by maternal drinking during pregnancy (yes, %) or maternal drinking amount (low or high). Logistic regression models were used to calculate adjusted odds ratios (ORs) and their 95% confidence intervals (CIs) after controlling simultaneously for potential covariates. Model covariates were based on previously published literature and biologic plausibility as potential covariates.^1,2,14–18^ Spearman’s rank correlation test was employed to check for multicollinearity. We then scrutinized the collection of final models based on Hosmer–Lemeshow Goodness-Of-Fit (HL-GOF) as one criterion of fit and the Akaike Information Criterion (AIC). We limited our analysis to male infants when employing logistic regression models for male genital abnormality.

## Results

**Fig. 1:**
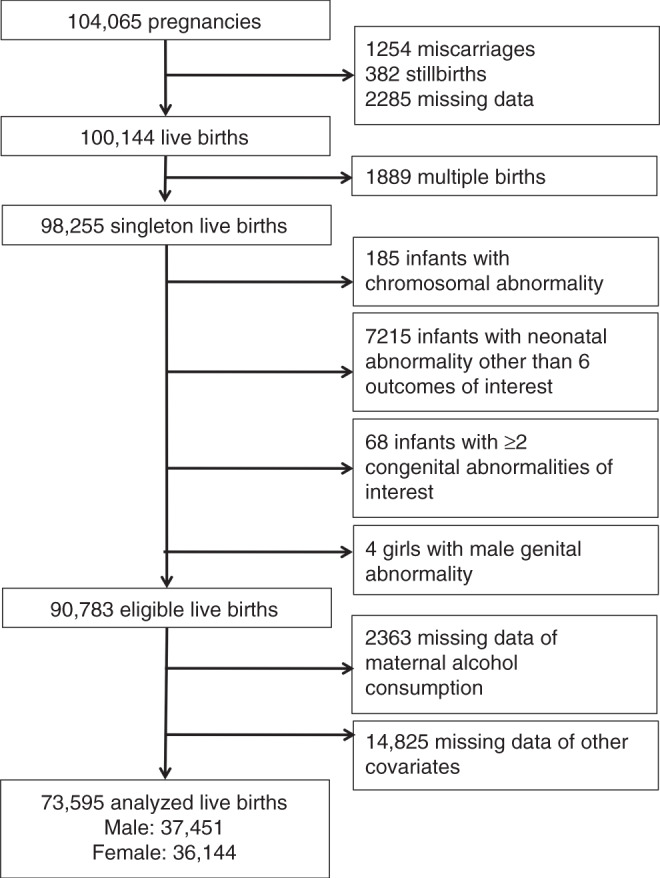
Participant eligibility flowchart.

In total, 73,595 mothers with singleton births and complete data acquisition were used for analysis (Fig. 1). The percentage of mothers who continued drinking until the second/third trimester was 2.8% (2,076/73,595), while that of mothers who quit drinking during early pregnancy was 46.6% (34,327/73,595) (Table 1). Supplemental Table S1 (online) summarizes the answers to questions on maternal drinking habits. Supplemental Table S2 (online) lists the distribution of respondents by drinking frequency and drinking amount. Many participants quit drinking early in pregnancy, with a small number continuing to drink until the second/third trimester of pregnancy. Of the subjects who continued to drink, 5.7% drank >5 times a week and 21% drank >1.5 drinks per week.

**Table 1:** Characteristics of participants with or without congenital malformation.

Variable	No congenital malformation	Congenital heart disease	Male genital abnormality	Limb defect	Cleft lip and/or cleft palate	Brain abnormality	Gastrointestinal obstruction	*P*
Participants, *n*	72,061	799	266	182	163	77	47	
Maternal age at delivery, years (mean ± SD)	31.3 ± 4.9	31.4 ± 5.1	31.9 ± 5.1	31.4 ± 4.9	30.9 ± 4.8	31.0 ± 5.5	31.7 ± 5.2	0.46^a^
Maternal age group, *n* (%)								0.61
<35 years	52,525 (72.9)	584 (73.1)	183 (68.8)	133 (73.1)	123 (75.5)	52 (67.5)	32 (68.1)	
35+ years	19,536 (27.1)	215 (26.9)	83 (31.2)	49 (26.9)	40 (24.5)	25 (32.5)	15 (31.9)	
Maternal BMI before pregnancy, kg/m^2^ (mean ± SD)	21.2 ± 3.3	21.5 ± 3.8	21.3 ± 3.5	21.7 ± 3.4	21.6 ± 4.0	21.1 ± 3.1	20.8 ± 3.2	0.07^a^
Maternal BMI group, *n* (%)								<0.001
Underweight (BMI <18.5)	11,354 (15.8)	153 (19.1)	43 (16.2)	26 (14.3)	29 (17.8)	8 (10.4)	12 (25.5)	
Normal weight (BMI 18.5–24.9)	53,085 (73.7)	534 (66.8)	188 (70.7)	128 (70.3)	108 (66.3)	60 (77.9)	33 (70.2)	
Overweight (BMI 25.0+)	7,622 (10.6)	112 (14.0)	35 (13.2)	28 (15.4)	26 (16.0)	9 (11.7)	2 (4.3)	
Highest level of education, *n* (%)								0.91
Junior high school	3,127 (4.3)	38 (4.8)	12 (4.5)	9 (4.9)	7 (4.3)	7 (9.1)	3 (6.4)	
High school	22,399 (31.1)	242 (30.3)	91 (34.2)	57 (31.3)	59 (36.2)	24 (31.2)	12 (25.5)	
Vocational school/Junior college	30,585 (42.4)	340 (42.6)	108 (40.6)	79 (43.4)	61 (37.4)	32 (41.6)	23 (48.9)	
University/Graduate school	15,950 (22.1)	179 (22.4)	55 (20.7)	37 (20.3)	36 (22.1)	14 (18.2)	9 (19.1)	
Annual household income, *n* (%)								0.95
<4,000,000 JPY	29,149 (40.5)	333 (41.5)	98 (36.8)	75 (41.2)	61 (37.4)	29 (37.7)	19 (40.4)	
4,000,000–7,999,999 JPY	35,230 (48.9)	390 (48.8)	141 (53.0)	92 (50.5)	82 (50.3)	39 (50.6)	23 (48.9)	
8,000,000+ JPY	7,682 (10.7)	76 (9.5)	27 (10.2)	15 (8.2)	20 (12.3)	9 (11.7)	5 (10.6)	
Maternal smoking during pregnancy, *n* (%)	3,142 (4.4)	40 (5.0)	16 (6.0)	12 (6.6)	7 (4.3)	5 (6.5)	2 (4.3)	0.49
Partner’s smoking during pregnancy, *n* (%)	33,530 (46.5)	370 (46.3)	123 (46.2)	92 (50.5)	76 (46.6)	32 (41.6)	20 (42.6)	0.89
Menstrual abnormality	13,107 (18.2)	161 (20.2)	52 (19.5)	39 (21.4)	36 (22.1)	20 (26.0)	7 (14.9)	0.19
Recurrent pregnancy loss, *n* (%)	716 (1.0)	10 (1.3)	3 (1.1)	2 (1.1)	1 (0.6)	3 (3.9)	0 (0.0)	0.25
Means of pregnancy for current birth, *n* (%)								0.01
Spontaneous	67,468 (93.6)	743 (93.0)	239 (89.8)	163 (89.6)	149 (91.4)	70 (90.9)	44 (93.6)	
Ovulation induction through medication	1,837 (2.5)	23 (2.9)	11 (4.1)	12 (6.6)	9 (5.5)	3 (3.9)	2 (4.3)	
Artificial insemination or in vitro fertilization	2,576 (3.8)	33 (4.1)	16 (6.0)	7 (3.8)	5 (3.1)	4 (5.2)	1 (2.1)	
Folic acid supplement, *n* (%)	1,573 (2.1)	24 (3.0)	3 (1.1)	5 (2.7)	4 (2.5)	1 (1.3)	1 (2.1)	0.57
Treatment for recurrent pregnancy loss, *n* (%)	3,494 (4.8)	43 (5.4)	16 (6.0)	6 (3.3)	7 (4.3)	5 (6.5)	4 (8.5)	0.66
Hypertensional disorder of pregnancy, *n* (%)	2,128 (3.0)	48 (6.0)	15 (5.6)	8 (4.4)	9 (5.5)	0 (0.0)	4 (8.5)	<0.001
Diabetes mellitus/gestational diabetes mellitus, *n* (%)	2,199 (3.1)	50 (6.3)	12 (4.5)	11 (6.0)	9 (5.5)	4 (5.2)	1 (2.1)	<0.001
Maternal infection during pregnancy, *n* (%)	15,734 (21.8)	172 (21.5)	63 (23.7)	43 (23.6)	31 (19.0)	11 (14.3)	11 (23.4)	0.63
Intrauterine fetal infection, *n* (%)	348 (0.5)	7 (0.9)	1 (0.4)	2 (1.1)	4 (2.5)	0 (0.0)	0 (0.0)	0.008
Maternal drinking during pregnancy, *n* (%)								0.30
Never drunk	36,359 (50.5)	438 (54.2)	143 (53.8)	96 (52.7)	90 (55.2)	46 (59.7)	20 (42.60)	
Quit drinking in early pregnancy	33,666 (46.7)	342 (42.8)	115 (43.2)	82 (45.1)	69 (42.3)	28 (36.4)	25 (53.2)	
Current drinking	2,036 (2.8)	19 (2.4)	8 (3.0)	4 (2.2)	4 (2.5)	3 (3.9)	2 (4.3)	
Maternal drinking amount of current drinkers, *n* (%)								0.44
Low (<1.5 drinks per week)	1,650 (2.3)	14 (1.8)	4 (1.5)	4 (2.2)	2 (1.2)	3 (3.9)	2 (4.3)	
High (1.5+ drinks per week)	386 (0.5)	5 (0.6)	4 (1.5)	0 (0.0)	2 (1.2)	0 (0.0)	0 (0.0)	

^a^Differences in maternal age and BMI were assessed with one-way repeated measures of ANOVA followed by post hoc (Bonferroni) testing.

The prevalence of CHDs, male genital abnormality, limb defect, OFC, brain abnormality, and gastrointestinal obstruction was 799 (1.1%), 266 (0.71% among male), 182 (0.25%), 163 (0.22%), 77 (0.10%), and 47 (0.06%) cases, respectively (Table 1). The participants’ characteristics including alcohol consumption and possible correlations with congenital malformations are summarized in Table 1. No remarkable differences were found between the presence of a congenital malformation and either maternal drinking during pregnancy or maternal drinking amount.

The adjusted OR estimates for maternal drinking habit and each type of congenital malformation are summarized in Table 2. We observed that the onset of CHD was inversely associated with mothers who quit drinking during early pregnancy (adjusted OR 0.85, 95% CI 0.74–0.98) and a negative trend among maternal drinking habits during pregnancy (*P* for trend = 0.023). There was no significant adverse impact or trend of maternal drinking habit status on the other congenital malformations after adjustment for covariates. HL-GOF and AIC both verified the fitness of the models used in the analysis (Table 2).

**Table 2:** Adjusted odds ratio estimates for maternal drinking habit status and each type of congenital malformation.

Variable	Congenital heart disease (*n* = 799)			Male genital abnormality (*n* = 266)			Limb defect (*n* = 182)			Cleft lip and/or cleft palate (*n* = 163)			Brain abnormality (*n* = 77)			Gastrointestinal obstruction (*n* = 47)		
	HL-GOF *P* = 0.960			HL-GOF *P* = 0.637			HL-GOF *P* = 0.985			HL-GOF *P* = 0.938			HL-GOF *P* = 0.107			HL-GOF *P* = 0.872		
	AICc = 460.5			AICc = 272.7			AICc = 243.6			AICc = 252.1			AICc = 132.9			AICc = 112.6		
	OR	95% CI	*P*	OR	95% CI	*P*	OR	95% CI	*P*	OR	95% CI	*P*	OR	95% CI	*P*	OR	95% CI	*P*
Drinking habit status																		
No drinking during pregnancy	Reference			Reference			Reference			Reference			Reference			Reference		
Quit drinking during early pregnancy	0.85	0.74–0.98	0.03	0.88	0.69–1.12	0.30	0.93	0.69–1.25	0.64	0.84	0.61–1.15	0.27	0.66	0.41–1.06	0.08	1.33	0.74–2.40	0.34
Current drinker	0.78	0.49–1.24	0.30	0.93	0.45–1.91	0.85	0.72	0.26–1.97	0.53	0.82	0.30–2.25	0.70	1.09	0.34–3.55	0.89	1.75	0.40–7.59	0.45

Model was adjusted for maternal age, BMI before pregnancy, maternal smoking habit, means of pregnancy, and complications during pregnancy (including diabetes mellitus/gestational diabetes mellitus, and hypertensive disorder of pregnancy, and intrauterine fetal infection).

*HL-GOF* Hosmer–Lemeshow goodness of fit, *AICc* Akaike’s information criterion with a correction for small sample size.

## Discussion

We herein describe the first nationwide birth cohort study in Japan to assess the effect of maternal alcohol consumption during pregnancy on major congenital malformations. The results of this large study indicated that alcohol consumption during pregnancy had no significant adverse impact on the prevalence of six categories of congenital malformations. In this survey of singleton live births, the prevalence of CHDs, male genital abnormality, limb defect, OFC, brain abnormality, and gastrointestinal obstruction were 1.1%, 0.71% among male infants, 0.25%, 0.22%, 0.10%, and 0.06%, respectively, and equivalent to those of previous studies,^19,20^ although they can vary by region and race.^21^

Although it is widely acknowledged that the etiology of congenital malformations can be multifactorial,^1,2^ it is also necessary to examine the independent effects of alcohol on birth defects. Among the organ systems affected by prenatal alcohol exposure, the brain is the most profoundly impacted, with reported reductions in brain volume and corpus callosum malformations.^22–24^ In this investigation, only the severe brain abnormalities of hydrocephalus, anencephaly, and holoprosencephaly were assessed to reveal no significant association with maternal alcohol consumption. It is important to note, however, that these results do not exclude the potential of milder brain abnormality or dysfunction from lower level of maternal alcohol consumption. This study suffered from a lack of data on brain volume and corpus callosum alterations since no imaging studies were carried out. Furthermore, congenital malformations were diagnosed up to 1 month after birth, during which time the assessment of brain function was difficult.

The effects of maternal drinking during pregnancy on congenital malformations other than the brain are controversial. Considerable attention has been given to the impact of fetal alcohol exposure on CHDs. The present study suggested that quitting drinking early in pregnancy might reduce the risk of CHDs, although continued drinking was not a significant risk factor for CHD occurrence. Yang et al. recently conducted a meta-analysis on the association between prenatal alcohol exposure and the risk of overall CHDs and reported no relationships with overall CHDs and some subtypes, with significant associations for conotruncal defects and dextro-transposition of the great arteries.^25^ Sun et al. also conducted a stratified analysis on the relationship of maternal alcohol consumption period, including pre-pregnancy and early pregnancy, with CHD risk in offspring.^6^ Their results indicated that drinking during early pregnancy, despite being a sensitive organogenesis period, was not related to the onset of CHDs. In the present nationwide survey, 2.8% of mothers reported alcohol consumption even after awareness of their pregnancy. Previous Japanese birth cohort studies have described higher drinking rates of 11.8%^26^ and 13.4%.^27^ Therefore, maternal alcohol consumption levels in this study may have been below the teratogenic threshold. Zhu et al. reported significantly or marginally significantly reduced risks for several CHD categories among mothers who reported to have drunk during pregnancy. This was especially evident for simple and high prevalence categories, including atrial septal defect, pulmonic valve stenosis, and aortic valve stenosis.^28^ They discussed the lack of increased risk for CHDs associated with maternal alcohol consumption to be attributable to the light or moderate levels of reported drinking. The inverse association in their case–control study was consistent with that in our investigation. Henderson et al. carried out a systematic review on the impact of low-to-moderate prenatal alcohol exposure (up to 10.4 UK units or 83 g/week) on pregnancy outcomes and detected no convincing evidence of adverse effects.^29^ However, the above studies suggested that heavy drinking and binge drinking during pregnancy were associated with overall CHD risk.^25^ Although other recent studies revealed negligible effects of light drinking on adverse birth outcomes,^29–33^ the true threshold of when alcohol constitutes a teratogen is unclear.^29^ We observed a negative trend among maternal alcohol drinking habits during pregnancy (*P* for trend = 0.023). Although mothers who quit drinking during early pregnancy were not asked about the amount of alcohol consumption in this survey, they were expected to have consumed a similarly low amount of alcohol during early pregnancy that was comparable to the “currently drinking” group. Wen et al. reported a J-shape dose–response curve correlation between the amount of alcohol consumption during pregnancy and the relative risk of CHDs in their meta-analysis.^34^ Similarly to this study, they showed that the corresponding risk of CHDs did not increase with the proportion of drinking amount. Further study is required to clarify the risk of congenital abnormalities according to the amount of alcohol consumption.

Fetal exposure to alcohol may disrupt cranial neural crest cells and result in the development of craniofacial structure anomalies, including brain damage and facial features^35^; some specific facial features, such as a smooth philtrum, thin upper lip, and small palpebral fissures, are often visible in individuals with fetal alcohol spectrum disorders.^36^ OFC also derive from cranial neural crest cell abnormalities, with a possible association with prenatal alcohol consumption.^37^ However, the results of this large study indicated that drinking during pregnancy did not remarkably increase the risk of OFC. Bell et al. conducted a systematic review examining the relationship between fetal alcohol exposure and the occurrence of OFC and detected no significant association as well.^5^ On the other hand, analysis of a large population-based study using data from the National Birth Defects Prevention Study (NBDPS) identified increased risks related to the amount, pattern, and type of alcohol consumed.^38^ The link between maternal alcohol consumption and OFC remains inconclusive.

Lastly, the precise association between fetal alcohol exposure and birth defects of the limbs and gastrointestinal tract is unknown. Limb defects are characterized by the failure of a part or the entire upper or lower limb to form during embryonic development. Previous studies implicated several risk factors with altered limb development, including maternal medication (thalidomide and vasoactive medications), health conditions (DM), and procedures received during pregnancy.^1,33^ Although experimental animal studies have uncovered possible mechanisms by which alcohol exposure during fetal development influences limb development,^39–43^ human epidemiological studies have failed to demonstrate any meaningful associations. Examination of data from the NBDPS showed an inverse association between maternal periconceptional drinking and a teratogen for selected limb defects, possibly due to under-reporting of maternal alcohol consumption,^33^ which was in agreement with our results.

This investigation had several limitations. First, the data about maternal alcohol consumption were obtained from self-reported questionnaires and depended on the validity and reliability of self-reported alcohol intake. Yokoyama et al.^11^ reported that a food frequency questionnaire provided reasonably valid measures for evaluating Japanese individuals for alcohol consumption. However, self-reports are often retrospective and may include response bias from the socially sensitive nature of the questions. Under-reporting of alcohol intake was also likely. Additionally, the exclusion of participants who did not respond to drinking as missing data may have constituted selection bias toward the null. Second, because this study detected very few frequent or heavy drinkers among respondents, a dose–response effect of alcohol consumption on congenital malformation development could not be assessed. Third, selection bias might have influenced the results based on the long-term cohort study design. Fourth, the study suffered from a lack of detailed information on the congenital malformation diagnoses because physicians transcribed only the presence of abnormalities at birth from medical files. Thus there may have been differences in the diagnostic criteria and degree of malformation^44^ leading to misclassification bias. In the JECS protocol, misclassifications were considered equivalent to environmental toxin exposure since the raters were blinded to assessments.^44^ Such misclassifications might have widened the 95% CI. Fifth, the incidence of congenital abnormalities can vary by racial background and demonstrates at least some familial heritability.^45,46^ Because of the racial differences in genetic factors related to alcohol metabolism as well as the onset of birth defects,^47^ the findings of this study may not be applicable to non-Japanese populations. Finally, higher drinking during pregnancy can cause spontaneous abortion and stillbirth.^48–50^ Cases of death during the fetal period also have congenital abnormalities, including heart defects, skeletal abnormalities, and brain abnormalities, such as anencephaly and neural tube defects.^51^ Since this study was aimed at live births, we have not been able to analyze cases of miscarriages or stillbirths. The exclusion of spontaneous abortion or stillbirth may have missed some of the outcomes of interest leading to a bias toward the null. Despite these limitations, however, this is the first study using a dataset from a Japanese nationwide birth cohort study to evaluate the effect of maternal alcohol consumption during pregnancy on congenital malformations after controlling for previously identified confounders.

In conclusion, this study provided important information on the impact of fetal alcohol exposure on congenital malformations. No teratogenic associations were found between maternal retrospective reports of alcohol during consumption and congenital malformations after adjustment for covariates. Some inverse associations were indicated, especially for the onset of CHD among mothers who quit drinking during early pregnancy. However, these findings must be interpreted with caution, first because this study lacked data concerning later-detected minor and asymptomatic defects, and second since it could not address the precise effects of heavy and binge drinking.

## Supplementary information

Supplementary Tables
