# Supplementary material for: Maternal alcohol consumption and risk of offspring with congenital malformation: the Japan Environment and Children’s Study
Source: Pediatr Res. 2020 Nov 23;90(2):479–86. doi: 10.1038/s41390-020-01274-9 (PMC8460444; doi:10.1038/s41390-020-01274-9)
Supplement: Supplementary file 1 — Supplementary Tables [file 41390_2020_1274_MOESM1_ESM.docx]

| **Supplemental table S1. Maternal drinking habits reported during the second/third trimester.** | | | | |
| --- | --- | --- | --- | --- |
| Answer content | | | | Number (%)* |
| Total no. | |  | | 90,783 |
| No answer to drinking habit | | | | 2363 (2.6) |
| No alcohol consumption | | | | 29656 (32.7) |
| Quit drinking before pregnancy | | | | 15166 (16.7) |
| Quit drinking during early pregnancy | | | | 41118 (45.3) |
| Currently drinking | | | | 2479 (2.7) |
|  | Frequency status | | |  |
|  |  | | Hardly drank | 691 (27.9) |
|  |  | | 1-3 times per month | 912 (36.8) |
|  |  | | 1-2 times per week | 395 (15.9) |
|  |  | | 3-4 times per week | 121 (4.9) |
|  |  | | 5-6 times per week | 68 (2.7) |
|  |  | | Daily | 75 (3.0) |
|  |  | | Missing data | 217 (8.8) |
|  | Amount status | | |  |
|  |  | | Low (<1.5 drinks per week) | 1788 (79.0) |
|  |  | | High (1.5+ drinks per week) | 475 (21.0) |
| *Number of subjects includes those with missing data. | | | |  |

| **Supplemental table S2. Drinking habit status of participants who reported "current drinking" during second/third trimester.** | | | | | |
| --- | --- | --- | --- | --- | --- |
| Drinking habit status | | | Total number (%) * | Amount status | |
|  |  |  |  | Low  (<1.5 drinks per week) | High  (1.5+ drinks per week) |
| Currently drinking | | | 2479 (2.7) | 1788 | 474 |
|  | Frequency status | |  |  |  |
|  |  | Hardly drank | 691 (27.9) | 691 | 0 |
|  |  | 1-3 times per month | 912 (36.8) | 880 | 32 |
|  |  | 1-2 times per week | 395 (15.9) | 204 | 191 |
|  |  | 3-4 times per week | 121 (4.9) | 7 | 114 |
|  |  | 5-6 times per week | 68 (2.7) | 4 | 64 |
|  |  | Daily | 75 (3.0) | 2 | 73 |
|  |  | Missing data | 217 (8.8) | NA | NA |

*Number of subjects includes those with missing data. NA, not applicable.
